# Novel Cardiovascular Risk Factors in Patients with Diabetic Kidney Disease

**DOI:** 10.3390/ijms222011196

**Published:** 2021-10-17

**Authors:** Christodoula Kourtidou, Maria Stangou, Smaragdi Marinaki, Konstantinos Tziomalos

**Affiliations:** 1 First Propedeutic Department of Internal Medicine, Medical School, Aristotle University of Thessaloniki, AHEPA Hospital, 54636 Thessaloniki, Greece; ktziomalos@yahoo.com; 2 Department of Nephrology, Medical School, Aristotle University of Thessaloniki, Hippokration Hospital, 54642 Thessaloniki, Greece; mstangou@auth.gr; 3 Department of Nephrology and Renal Transplantation, Medical School, National and Kapodistrian University of Athens, Laiko Hospital, 11527 Athens, Greece; smarinak@med.uoa.gr

**Keywords:** diabetes mellitus, diabetic kidney disease, cardiovascular risk, neutrophil gelatinase-associated lipocalin, kidney injury molecule-1, lipoxygenases, copeptin, matrix metalloproteinases, fibroblast growth factor-23, klotho, cubilin

## Abstract

Patients with diabetic kidney disease (DKD) are at very high risk for cardiovascular events. Only part of this increased risk can be attributed to the presence of diabetes mellitus (DM) and to other DM-related comorbidities, including hypertension and obesity. The identification of novel risk factors that underpin the association between DKD and cardiovascular disease (CVD) is essential for risk stratification, for individualization of treatment and for identification of novel treatment targets.In the present review, we summarize the current knowledge regarding the role of emerging cardiovascular risk markers in patients with DKD. Among these biomarkers, fibroblast growth factor-23 and copeptin were studied more extensively and consistently predicted cardiovascular events in this population. Therefore, it might be useful to incorporate them in risk stratification strategies in patients with DKD to identify those who would possibly benefit from more aggressive management of cardiovascular risk factors.

## 1. Introduction

Diabetic nephropathy is the commonest chronic kidney disease (CKD) [1]. Several studies consistently showed that patients with diabetic kidney disease (DKD) have increased cardiovascular risk [2,3]. Indeed, patients with DKD have threetimes higher all-cause mortality and a 16-year loss in life expectancy compared with the general population [2]. Moreover, patients with DKD appear to have a similar or even higher incidence of cardiovascular events compared with patients with coronary heart disease [3]. Even though both diabetes mellitus (DM) per se and DM-related comorbidities, including hypertension and obesity, are established cardiovascular risk factors, they do not fully explain the higher cardiovascular morbidity in patients with DKD [2,3]. The identification of novel risk factors that underpin the association between DKD and cardiovascular disease (CVD) is essential for risk stratification, individualization of treatment and for identification of novel treatment targets.

In the present review, we summarize the current knowledge regarding the role of emerging cardiovascular risk factors in patients with DKD.

## 2. Neutrophil Gelatinase-Associated Lipocalin

Neutrophil gelatinase-associated lipocalin (NGAL) is a polypeptide that is secreted by injured kidney tubular epithelial cells [4]. Patients with diabetic nephropathy have higher NGAL levels than healthy controls [5,6,7]. In addition, NGAL levels correlate with glomerular filtration rate (GFR) and urinary albumin excretion [7,8,9,10,11,12]. Moreover, NGAL levels predict GFR decline and progression to end-stage renal disease (ESRD) in patients with DKD [13,14,15,16].

A number of small studies evaluated the association between NGAL levels and cardiovascular events in patients with DKD yielding mostly negative results. In 91 elderly men with T2DM, urinary NGAL levels did not predict cardiovascular mortality [17]. In another prospective study in 200 patients with type 2 DM (T2DM) and persistent microalbuminuria followedup for 6.1 years, higher urinary NGAL levels did not predict either GFR decline or cardiovascular events [18]. In contrast, in a prospective study in 5380 patients with T2DM and a recent acute coronary syndrome, NGAL levels predicted the composite endpoint of nonfatal myocardial infarction (MI), nonfatal stroke and cardiovascular death independently of GFR [19].

## 3. Kidney Injury Molecule 1

Kidney injury molecule-1 (KIM-1) is a type 1 epithelial transmembrane glycoprotein, and its expression is upregulated in the proximal tubules of the kidney following an ischemic insult [20]. Patients with diabetic nephropathy have higher KIM-1 levels than controls [5,21,22,23]. Moreover, KIM-1 levels correlate with GFR and urinary albumin excretion [5,8,24,25,26]. Elevated KIM-1 levels also predict the onset of microalbuminuria [27], a decline in GFR and the incidence of ESRD in patients with either T2DM or type 1 DM (T1DM) [13,28,29,30].

It appears that KIM-1 levels represent a promising cardiovascular risk marker in patients with DKD. Indeed, in a small study in 91 elderly men with T2DM, urinary KIM-1 levels independently predicted cardiovascular mortality [17]. In a larger prospective study in 5380 patients with T2DM and a recent acute coronary syndrome, KIM-1 levels predicted the composite endpoint of nonfatal MI, nonfatal stroke and cardiovascular death independently of GFR [19]. More importantly, in a prospective study in 200 patients with T2DM and persistent microalbuminuria followed-up for 6.1 years, higher urinary KIM-1 levels predicted both GFR decline and cardiovascular events [18]. In contrast, in another study in 231 patients with T2DM and CKD who were followedup for 7 years, KIM-1 levels did not predict cardiovascular events [31].

## 4. Lipoxygenases

Lipoxygenases are a family of enzymes that metabolize polyunsaturated fatty acids into active products that promote inflammation and oxidative stress [32,33,34,35]. Preclinical studies showed that 12-lipooxygenase promotes fibrogenesis in the kidneys of patients with T2DM both directly and by augmenting the effects of angiotensin II [36,37,38]. Patients with diabetic nephropathy have higher levels of products of 12-lipoxygenase than patients with T2DM but without nephropathy [39,40]. Moreover, polymorphisms in the 5- and 12-lipoxygenase genes are associated with diabetic nephropathy and more pronounced albuminuria in patients with T2DM, respectively [41,42].

Preliminary data suggest a relationship between lipoxygenase activity and atherosclerosis in patients with DKD. In a small study in 145 patients with T2DM and CKD, polymorphisms of the 12-lipoxygenase gene were associated with greater carotid intima-media thickness (cIMT), a marker of subclinical atherosclerosis, and with a higher incidence of cardiovascular events and cardiovascular mortality during a 7-year follow-up period [43]. In the Diabetes Heart Study (828 diabetic and 170 non-diabetic siblings), polymorphisms in the same gene were also associated with subclinical atherosclerosis (coronary, carotid and aortic calcification as well as cIMT) [44].

## 5. Copeptin

Copeptin is the C-terminal portion of pre-provasopressin and a surrogate marker of vasopressin levels, since it is more stable and more easily measured than vasopressin and correlates strongly with vasopressin concentration [45,46]. Copeptin levels are elevated in patients with DM [47,48], possibly due to a glycosuria-associated reduction of extracellular volume and a reset of receptors that regulate vasopressin secretion [40,49]. In animal models of DM, vasopressin was shown to promote hyperfiltration and albuminuria [50,51,52]. In patients with T1DM, copeptin is associated with intrarenal activation of the renin-angiotensin system (RAS) and with increased renal vascular resistance [53]. Several cross-sectional studies reported a correlation between plasma copeptin levels and both GFR and urinary albumin excretion [54,55,56,57]. In the prospective DIABHYCAR study (*n* = 3101 patients with T2DM and albuminuria), plasma copeptin levels independently predicted the doubling of serum creatinine levels or development of ESRD during a 6-year follow-up period [58]. In the Zwolle Outpatient Diabetes project Integrating Available Care (ZODIAC) cohort (756 patients with T2DM followed-up for 6.5 years), plasma copeptin levels were also associated with a decline in GFR but only in patients not using RAS inhibitors [54]. In a smaller study, the Skaraborg Diabetes Register (*n* = 161 patients with newly diagnosed T2DM), plasma copeptin levels also independently predicted GFR decline during a 12-year follow-up [59].

Accumulating evidence supports the role of copeptin in cardiovascular risk prediction in patients with DKD. In patients with T1DM, copeptin levels positively correlated with the severity of arterial stiffness [60] and with coronary artery calcification, a marker of subclinical atherosclerosis [56]. More importantly, iIn the DIABHYCAR study (*n* = 3101 patients with T2DM and albuminuria) and in the SURDIAGENE cohort (*n* = 1407 patients with T2DM), plasma copeptin levels were associated with increased risk of cardiovascular events during a median follow-up of 5 years [61]. In the ZODIAC cohort (1195 patients with T2DM followed-up for 5.9 years), plasma copeptin levels also predicted cardiovascular mortality [62]. In a smaller study with a shorter follow-up (*n* = 781 patients with T2DM followed-up for 15 months), copeptin levels were higher in patients who experience a cardiovascular event but this association was not significant in multivariate analysis [63]. In two cohorts of patients with T1DM followedup for 10.2 and 5 years, respectively (*n* = 398 and 588, respectively), plasma copeptin levels were associated with both a higher incidence of ESRD and with a higher risk of MI or coronary revascularization [64].

## 6. Matrix Metalloproteinases

Matrix metalloproteinases (MMPs) are a family of zinc-dependent endoproteases with multiple roles in tissue remodeling [65,66]. In cross-sectional studies in patients with T2DM, impaired kidney function was associated with higher urine levels of MMP-9 [67] and higher serum levels of MMP-10 and -2 [68,69]. In another study including 75 patients with T2DM, urinary MMP-9 levels were higher in patients with T2DM compared with healthy subjects and patients with T2DM and albuminuria had higher MMP-9 levels than patients with T2DM but without albuminuria [70]. In a cross-sectional study with data from the EURODIAB Prospective Complications Study (*n* = 493 patients with T1DM), higher plasma levels of MMP-2, MMP-3 and MMP-10 were associated with macroalbuminuria [71]. In a prospective study (*n* = 1181 patients with T2DM and GFR ≥ 60 mL/min/1.73m^2^ followed-up for 6–12 years), increased circulating levels of MMP-7 were linked with early progressive renal decline, defined as annual GFR loss of ≥ 5 mL/min/1.73 m^2^/year [72].

Recent data support an association between MMP levels and CVD in patients with DKD. In two cohorts of patients with T2DM with DKD and cardiac diastolic dysfunction (*n* = 60 and 40, respectively), serum MMP-7 level was elevated in both groups [73]. In a study including data from three different cohorts of patients with T1DM, namely EURODIAB Prospective Complications Study (*n* = 509), LEACE (*n* = 370) and PROFIL (*n* = 638), serum MMP-1, -2 and -3 levels correlated with the severity of arterial stiffness [74]. In the SUMMIT cohort (*n* = 985 subjects with T2DM and 515 controls), plasma levels of MMP-7 and MMP-12 were increased in patients with T2DM and were higher in patients with T2DM and CVD than in those without CVD [75]. In a study in 1090 patients with T2DM, the T allele of MMP-2 C (−1306)T polymorphism was associated with a lower risk of CVD and lower susceptibility to stroke [76].

## 7. Fibroblast Growth Factor-23

Fibroblast growth factor-23 (FGF-23) is a hormone that plays an important role in vitamin D and phosphate homeostasis [77]. In patients with T2DM and CKD, increased serum FGF-23 levels were associated with macroalbuminuria and creatinine levels [77,78,79,80]. In addition, serum FGF-23 levels predicted an increased risk for DKD progression [81].

Several studies showed that FGF-23 is also associated with increased risk for CVD in patients with DKD. In a cross-sectional study in 71 patients with T1DM and early DKD, FGF-23 levels correlated with diastolic cardiac dysfunction [82]. In a larger cross-sectional study in 246 patients with Τ2DM, increased serum levels of FGF-23 were also associated with cardiac diastolic dysfunction and with reduced myocardial perfusion reserve [83]. In another cross-sectional study in 545 African American patients with T2DM, FGF-23 concentrations were associated with the extent of coronary artery calcification [84]. In a cohort study (*n* = 1211 patients with T2DM), increased FGF-23 levels independently predicted incident cardiovascular events [85]. In the prospective DIALECT study (*n* = 310 patients with GFR > 60 mL/min/1.73 m^2^), elevated plasma FGF23 levels were associated with increased risk for cardiovascular morbidity and mortality [86]. In another prospective study (*n* = 380 patients with T2DM followed-up for 8–12 years), plasma FGF-23 levels were associated with greater cardiovascular mortality [87]. In a prospective study in 107 T2DM patients with stage 2–3 CKD, higher serum FGF-23 levels were associated with increased risk for hospitalization for cardiovascular events and higher cardiovascular mortality [88].

## 8. Klotho

Klotho is a transmembrane protein that forms co-receptors with FGF-23 receptors to enhance the binding of FGF-23 [89]. In two studies, lower serum levels of α-Klotho and β-Κlotho were found in patients with T2DM compared with healthy subjects [90,91]. A negative correlation was also identified between serum α-Klotho and the development of albuminuria in T2DM patients [90]. In cross-sectional studies, serum Klotho levels were associated with urinary albumin to creatinine ratio [92,93,94]. In a cohort (*n* = 63 patients with diabetic kidney disease) high levels of serum s-Klotho were associated with faster progression of CKD [95]. In a study (*n* = 101 patients with T2DM and eGFR > 45 mL/min), lower s-Klotho levels were correlated with a faster rate of decline in eGFR as compared with higher levels during a median follow-up of 9 years [96]. In a prospective study (*n* = 107 patients with T2DM and Stage 2–3 CKD), low serum α-Klotho levels were associated with cardiac hypertrophy and a high risk of cardiovascular hospitalization and cardiovascular mortality [88].

## 9. Cubilin

Cubilin is an extracellular protein coexpressed with megalin in the proximal tubule and in podocytes [97]. Patients with T1DM and microalbuminuria have a more abundant expression of cubilin in the proximal tubule than both healthy controls and patients with T1DM and normoalbuminuria [98]. In a meta-analysis of genome-wide association studies in 5825 patients with DM and 46,061 controls, polymorphisms in the gene encoding cubilin were associated with urinary albumin excretion [99]. In a smaller study (*n* = 472 patients with T2DM) cubilin gene variants were associated with increased risk for both ESRD and peripheral arterial disease [100].

## 10. Non-Coding RNAs

MicroRNAs (miR) are non-coding, single-stranded RNA molecules containing 17–25 nucleotides that post-transcriptionally regulate their target genes by degradation or translational repression of the complementary messenger RNAs (mRNAs) [101]. It was reported that miR-126 is a marker of coronary heart disease in patients with T2DM [102].

Long non-coding RNAs (LncRNAs) also appear to be useful markers of cardiovascular risk [103]. It was shown that LncRNAs predict ESRD in patients with T1DM [104]. Moreover, in a meta-analysis of 30 studies, LncRNAs had good sensitivity and specificity in differentiating between patients with CVD and controls [105].

Circular RNAs (circRNAs) are another class of non-coding RNAs that also appears to play a role in the pathogenesis of DKD [106,107]. In addition, preliminary data suggest that circRNAs are independent predictors of MI [108].

## 11. Conclusions

**Figure 1:**
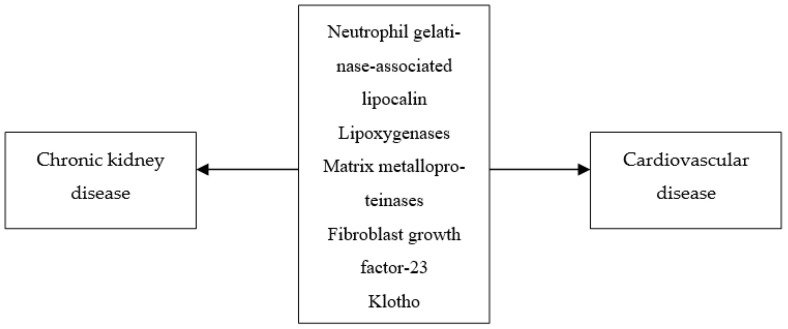
Factors associated with both chronic kidney disease and cardiovascular diseases in diabetic patients.

Several novel biomarkers appear to be independently associated with both renal damage and increased cardiovascular risk in patients with DKD (Table 1 and Table 2, Figure 1). Among these biomarkers, FGF-23 and copeptin were studied more extensively and consistently predicted cardiovascular events in this population. Therefore, it might be useful to incorporate them in risk stratification strategies in patients with DKD to identify those who would possibly benefit from more aggressive management of cardiovascular risk factors.

## Figures and Tables

**Table 1:** Novel biomarkers associated with kidney damage in patients with diabetic kidney disease.

Biomarker	Correlates with Glomerular Filtration Rate	Correlates with Urinary Albumin Excretion	Predicts Decline in Glomerular Filtration Rate
Neutrophil gelatinase-associated lipocalin	Yes	Yes	Yes
Kidney injury molecule-1	Yes	Yes	Yes
Lipoxygenases	Yes	Yes	Unknown
Copeptin	Yes	Yes	Yes
Matrix metalloproteinases	Yes	Yes	Yes
Fibroblast growth factor-23	Yes	Yes	Yes
Klotho	Yes	Yes	Yes
Cubilin	Yes	Yes	Yes

**Table 2:** Novel biomarkers associated with increased cardiovascular risk in patients with diabetic kidney disease.

Biomarker	Predicts Myocardial Infarction	Predicts Ischemic Stroke	Predicts Cardiovascular Mortality
Neutrophil gelatinase-associated lipocalin	Yes	Yes	Conflicting results
Kidney injury molecule-1	Conflicting results	Conflicting results	Conflicting results
Lipoxygenases	Yes	Yes	Yes
Copeptin	Conflicting results	Conflicting results	Conflicting results
Matrix metalloproteinases	Yes	Yes	Unknown
Fibroblast growth factor-23	Yes	Yes	Yes
Klotho	Yes	Yes	Yes
Cubilin	Unknown	Unknown	Unknown
